# Longitudinal Detection of Twenty DNA and RNA Viruses in Allogeneic Hematopoietic Stem Cell Transplant Recipients Plasma

**DOI:** 10.3390/v15040928

**Published:** 2023-04-07

**Authors:** Marie-Céline Zanella, Diem-Lan Vu, Krisztina Hosszu-Fellous, Dionysios Neofytos, Chistian Van Delden, Lara Turin, Antoine Poncet, Federico Simonetta, Stavroula Masouridi-Levrat, Yves Chalandon, Samuel Cordey, Laurent Kaiser

**Affiliations:** 1 Division of Infectious Diseases, Geneva University Hospitals, 1211 Geneva, Switzerland; 2 Laboratory of Virology, Division of Laboratory Medicine, Geneva University Hospitals, 1211 Geneva, Switzerland; 3 Faculty of Medicine, University of Geneva Medical School, 1206 Geneva, Switzerland; 4 Geneva Centre for Emerging Viral Diseases, 1211 Geneva, Switzerland; 5 Center for Clinical Research, Department of Health and Community Medicine, University of Geneva, 1206 Geneva, Switzerland; 6 Division of Clinical Epidemiology, Department of Health and Community Medicine, University Hospital of Geneva, 1211 Geneva, Switzerland; 7 Division of Hematology, Department of Oncology, Geneva University Hospitals, 1211 Geneva, Switzerland

**Keywords:** virus, virome, PCR, transplantation, anellovirus, TTV, human pegivirus, polyomavirus, blood

## Abstract

Metagenomics revealed novel and routinely overlooked viruses, representing sources of unrecognized infections after allogeneic hematopoietic stem cell transplantation (allo-HSCT). We aim to describe DNA and RNA virus prevalence and kinetics in allo-HSCT recipients’ plasma for one year post HSCT. We included 109 adult patients with first allo-HSCT from 1 March 2017 to 31 January 2019 in this observational cohort study. Seventeen DNA and three RNA viral species were screened with qualitative and/or quantitative r(RT)-PCR assays using plasma samples collected at 0, 1, 3, 6, and 12 months post HSCT. TTV infected 97% of patients, followed by HPgV-1 (prevalence: 26–36%). TTV (median 3.29 × 10^5^ copies/mL) and HPgV-1 (median 1.18 × 10^6^ copies/mL) viral loads peaked at month 3. At least one *Polyomaviridae* virus (BKPyV, JCPyV, MCPyV, HPyV6/7) was detected in >10% of patients. HPyV6 and HPyV7 prevalence reached 27% and 12% at month 3; CMV prevalence reached 27%. HSV, VZV, EBV, HHV-7, HAdV and B19V prevalence remained <5%. HPyV9, TSPyV, HBoV, EV and HPg-V2 were never detected. At month 3, 72% of patients had co-infections. TTV and HPgV-1 infections were highly prevalent. BKPyV, MCPyV and HPyV6/7 were frequently detected relative to classical culprits. Further investigation is needed into associations between these viral infections and immune reconstitution or clinical outcomes.

## 1. Introduction

Primary viral infections and reactivations are the most common infectious complications following allogeneic hematopoietic stem cell transplantation (allo-HSCT), and are often associated with significant morbidity and mortality [1,2]. These viral infections may be associated with transient viremia or protracted replication, and can range from asymptomatic to severe disease. The type and number of viral infections detected is closely linked to the detection methods used. The unbiased approach of metagenomic next-generation sequencing (mNGS) has provided a better understanding of viral infections and expanded our knowledge of the blood virome beyond the usual culprits such as Epstein–Barr virus (EBV), cytomegalovirus (CMV) or adenovirus (HAdV). Indeed, torque teno virus (TTV) and human pegivirus-1 (HPgV-1) are now known to be highly prevalent among HSCT recipients, while several novel *Polyomaviridae* have been detected in various clinical samples [3,4,5]. However, unlike real-time (RT-)PCR (r(RT-)PCR) methods, mNGS technology is currently marginally used in routine and often as a method of last resort. Moreover, mNGS is not yet a sufficiently reproducible method to perform unbiased quantitative analyses for the monitoring of numerous and specific viral species.

Most studies investigated only specific viruses [4], nevertheless, mNGS revealed that the co-detection of DNA and RNA viruses in blood is common after HSCT [3,5,6]. These viruses may be associated with yet unrecognized clinical manifestations and complex alterations of the immune system after HSCT. Furthermore, the blood virome composition changes with time after transplantation and the dynamics of viral infections after HSCT have only been reported in a few longitudinal studies using (RT)-PCR assays, mostly targeting DNA viruses or mNGS [3,4,5,6,7].

More data are needed on DNA and RNA virus infections after allo-HSCT using r(RT-)PCR assays, as they are routine, accessible and ready-to-use diagnostic tools for clinicians. For the purpose of this study, we selected seventeen DNA and three RNA viruses that are already characterized as pathogens or novel viruses detected in blood samples of transplant recipients that are potential causes of unrecognized infections in the context of intense immunosuppression [4]. The objective was to describe the prevalence and dynamics of infections by these DNA and RNA viruses using r(RT-)PCR assays on plasma samples of adult allo-HSCT recipients collected over a one-year period after allo-HSCT. The viral load (VL) in plasma was described using quantitative r(RT)-PCR assays.

## 2. Materials and Methods

This observational study was conducted at the Geneva University Hospitals (HUG), Switzerland. The study was approved by the Geneva Cantonal Ethics Committee (project #2017-01304). Data have been deposited to Dryad (https://doi.org/10.5061/dryad.5qfttdz81, accessed on 5 April 2023).

### 2.1. Patients and Samples

This is a longitudinal observational cohort study. Adult (≥18-year-old) patients that received a first allo-HSCT from 1 March 2017 to 31 January 2019 at HUG with a signed informed consent form to enroll in the local monocentric “infectious disease cohort of allo-HSCT patients” (project #CCER_15-120) were included. Pediatric patients and those who did not sign an informed written consent were excluded. Plasma samples were prospectively collected, for the purpose of the local cohort, for all included patients at pre-specified time points before and during a one-year period after allo-HSCT: seven days before transplantation (D-7), the day of transplantation before infusion of stem cells (D0), and 30 days (D30), 3 months (M3), 6 months (M6) and one year after transplantation (Y1). Plasma samples were stored in the Laboratory of Virology (HUG).

### 2.2. Antiviral Prophylaxis, Treatment and Routine Screening

According to institutional practice guidelines, patients received (val-)acyclovir as prophylaxis for herpes simplex virus (HSV) 1–2 and varicella zoster virus (VZV), from the first day of conditioning to up to 2 years post HSCT in specific situations (i.e., acute graft-versus-host disease (aGVHD) ≥grade 2 or moderate/severe chronic GVHD (cGVHD); additional immunosuppressive treatments). EBV and CMV DNA detection were monitored weekly by specific rPCR in plasma samples of all patients from conditioning to 100 days post HSCT or longer in specific cases. Since May 2019, primary CMV prophylaxis with oral letermovir was administered in CMV donor-negative and recipient-positive patients at day 1 post HSCT until day 100, and in CMV recipient-positive patients with acute GVHD ≥grade 2 receiving ≥1 mg/kg/day of prednisone equivalent [8]. Clinical testing and treatment of viral infections were performed according to international and institutional practice guidelines.

### 2.3. Virological Testing

**Figure 1:**
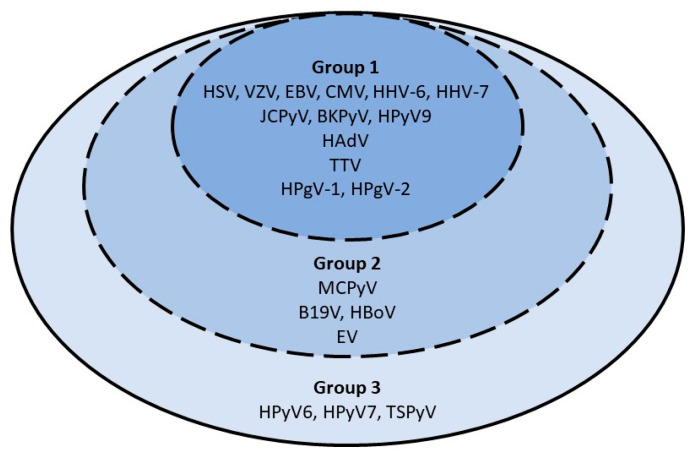
Priority order for virological testing on plasma samples. Patients in group 3 had screening for all 20 viral species (comprising viral species of groups 1, 2 and 3). Patients in group 2 were screened for 17 viral species (comprising viral species of group 1 and 2). Patients in group 1 were screened for 13 viral species only. Abbreviations: HSV-1/2: herpes simplex virus; VZV: varicella zoster virus; EBV: Epstein–Barr virus; CMV: cytomegalovirus: HHV-6A/B: human herpesvirus 6A/B; HHV-7: human herpes virus 7; JCPyV: JC polyomavirus; BKPyV: BK polyomavirus; MCPyV: Merkel cell polyomavirus; HPyV6: human polyomavirus 6; HPyV7: human polyomavirus 7; TSPyV: trichodysplasia spinulosa-associated polyomavirus; HPyV9: human polyomavirus 9; HAdV: human adenovirus; B19V: parvovirus B19; HBoV: human bocavirus; TTV: torque teno virus; EV: enterovirus; HPgV-1: human pegivirus 1; HPgV-2: human pegivirus 2.

A total of 17 DNA and 3 RNA viruses were screened with qualitative and/or quantitative r(RT)-PCR assays performed on plasma samples collected at D0, D30, M3, M6 and Y1 (Table S1). When plasma samples were unavailable at D0, plasma samples collected at D-7 were used. Considering the variable and limited amount of plasma samples, viruses were screened according to a pre-specified order of priority (Figure 1) and qualitative assays took precedence over quantitative assays.

### 2.4. Nucleic Acid Extraction and r(RT-)PCR Screening

Table S2 provides a detailed list of r(RT-)PCRs used. For all virus testing, except EBV and CMV, plasma was spiked with a standardized canine distemper virus (CDV) as an internal control [9]. Thereafter, nucleic acids were extracted using the NucliSENS easyMAG (bioMérieux, Geneva, Switzerland) nucleic acid kit, according to the manufacturer’s instructions. For patients in group 3, 1.6 mL of plasma was extracted with a final elution volume of 200 μL. For patients in group 2, 1.2 mL of plasma was extracted with a final elution volume of 150 μL. For patients in group 1, 1 mL of plasma was extracted with a final elution volume of 125 μL (alternatively, in case of very limited plasma volume, 0.8 mL of plasma in a final elution volume of 100 μL). For enterovirus (EV), HPgV-1 and -2, and CDV, rRT-PCRs were performed using the one-step Quanti-Tect Probe RT-PCR Kit (Qiagen, Hombrechtikon, Switzerland) in a StepOne Plus instrument (Applied Biosystems, Rotkreuz, Switzerland) under the following cycling conditions: 50 °C for 30 min, 95 °C for 15 min, 45 cycles of 15 s at 94 °C and 1 min at 55 °C (HPgV-1 and -2 and CDV) or 60 °C (EV). BK polyomavirus (BKPyV), parvovirus B19 (B19V), JC polyomavirus (JCPyV) quantitative, and HAdV quantitative PCRs were run in a StepOne Plus instrument according to the manufacturer’s instructions. HSV-1/2 typing PCR was performed using the TaqMan Universal PCR mastermix (Applied Biosystems) in a StepOne Plus instrument (Applied Biosystems) under the following cycling conditions: 50 °C for 2 min, 95 °C for 15 min, 45 cycles of 15 s at 95 °C and 1 min at 60 °C.

Screening rPCRs for HSV-1/2, VZV, human herpes virus 6 (HHV-6), JCPyV, HAdV, TTV, human polyomavirus (HPyV) 6/7/9, human bocavirus (HBoV) 1-4, Merkel cell polyomavirus (MCPyV), and trichodysplasia spinulosa-associated polyomavirus (TSPyV) were performed using the TaqMan Universal PCR mastermix (Applied Biosystems) in a QuantStudio 5 Real-Time PCR System (Applied Biosystems) under the following cycling conditions: 50 °C for 2 min, 95 °C for 15 min, 45 cycles of 15 s at 95 °C and 1 min at 55 °C (MCPyV), 58 °C (TSPyV) or 60 °C (all except MCPyV and TSPyV). HHV-6 quantitative and HHV-7 were also run in a QuantStudio 5 Real-Time PCR System instrument according to the manufacturer’s instructions.

Quantitative commercial diagnostic methods were used as first-line tests for EBV, CMV, HHV-7, BK and B19. For the other viruses, the viral loads were either directly estimated by reporting Ct values on standard curves previously obtained from 10-fold serial dilutions of specific plasmids or plasmid-derived transcribed RNA (i.e., for DNA and RNA viruses, respectively) including the target region, or, for HAdV, JCPyV and HHV-6, by testing only positive samples obtained with the first-line qualitative assay with a quantitative commercial diagnostic assay.

EBV and CMV results were obtained from routine clinical testing when available, or from plasma samples collected for the cohort. The commercial diagnostic methods used for routine testing of both viruses changed during our study investigations. Until 16 May 2018 CMV testing was performed using the RealTime CMV assay (Abbott) on the m2000 system (Abbott), and afterwards it was performed using the cobas^®^ CMV tests (Roche, Rotkreuz, Switzerland), on the cobas^®^ 6800 System (Roche). As the cobas^®^ CMV assay has a lower limit of quantification (LLOQ) than the RealTime CMV assay, a threshold of 5.6 × 10^1^ IU/mL was used. Similar to CMV, EBV testing was performed until 5 December 2019 using the artus EBV QS-RGQ kit (Qiagen) on nucleic acids extracted on a Qiasymphony instrument. Afterwards, it was performed using the cobas^®^ EBV tests on the cobas^®^ 6800 System. As the cobas^®^ EBV assay shows a LLOQ than the first assay, a threshold of 500 copies/mL was used for our analysis.

### 2.5. Statistical Methods

Patients’ characteristics were described as counts and percentages for qualitative variables, and as mean with standard deviation or median and interquartile range for quantitative variables. The prevalence of plasma detection of each virus was described over the one-year time period (from D0 to Y1; period prevalence) and at each time-point (point prevalence). The median VL and quartiles were described at each time-point for each virus that was detected in five or more patients and with VLs equal to or greater than the threshold of the r(RT-)PCR assay. Co-infections were investigated in patients for whom the 20 viruses could be tested. All statistical analyses were performed on R software version R-4.0.2 (R Foundation for Statistical Computing, Vienna, Austria. URL https://www.R-project.org/, accessed on 5 April 2023).

## 3. Results

### 3.1. Patient Characteristics

A total of 109 consecutive adult allo-HSCT recipients were included in the study. Patient characteristics are detailed in Table 1. At one year, 84 (79%) allo-HSCT recipients presented a GVHD (*n* = 77 aGVHD, including 39 grade ≥2; *n* = 31 cGVHD), 38 (35%) were deceased (including 26 relapse-associated deaths). The non-relapse mortality was 11%.

Three pre-specified groups of viruses are presented in Figure 1.

The number of patients for whom virological testing was performed at each time-point according to these groups is presented in Table 2.

### 3.2. Virological Testing

#### 3.2.1. Period Prevalence

From D0 to Y1, TTV was detected in 104 (95%) patients. BKPyV and HPgV-1 followed with detection in 52% and 47% of patients, respectively. Seven viruses (EBV, CMV, HHV-6, JCPyV, MCPyV, HPyV6 and HPyV7) were detected in 10% to 40% of the patients. Five viruses (HSV, VZV, HHV-7, HAdV and B19V) were detected in 2% to 5% of the patients, and five viruses were never detected (HPg-V2, HBoV, EV, HPyV9 and TSPyV) (Table 3).

Screening was not performed due to the following reasons: the viral species was not included in the pre-specified group of testing, insufficient volume of plasma, plasma sample not collected at the time of visit, missed visit, or patient’s death.

#### 3.2.2. Point Prevalence and Dynamic Evolution of Prevalence

Detailed results of virological testing for each viral species at each time-point are presented in Table 4.

**Figure 2:**
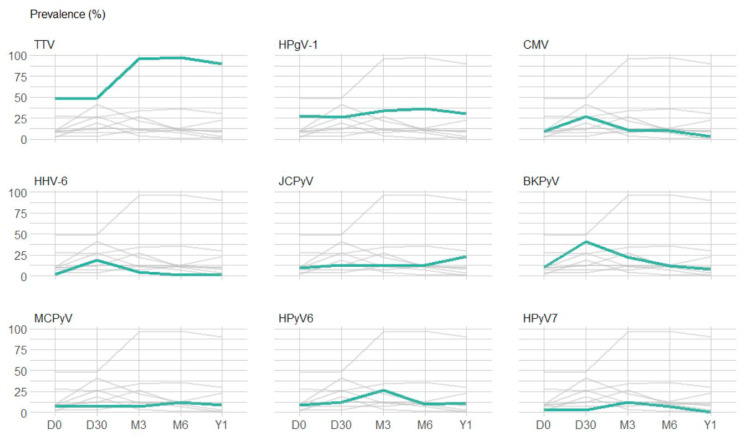
Prevalence of plasma detection of the 9 viruses with a plasma detection prevalence above 10%. The graphs represent the prevalence of the 9 viruses with a prevalence of plasma detection above 10% at least at one time-point after transplantation. For each graph, green lines highlight the prevalence of plasma detection of one specific virus and grey lines represent the prevalence of plasma detection of the other 8 viruses. Abbreviations: TTV: torque teno virus; HPgV-1: human pegivirus 1; HPyV6: human polyomavirus 6; BKPyV: BK polyomavirus; JCPyV: JC polyomavirus; HPyV7: human polyomavirus 7; CMV: cytomegalovirus; MCPyV: Merkel cell polyomavirus; HHV-6: human herpesvirus 6.

Figure 2 highlights the prevalence over time of frequently detected viruses (those with a prevalence ≥ 10% at one or more time-points). Before the infusion of stem cells (D0), 13 of the 20 screened viruses were detected (TTV, HPgV-1, JCPyV, BKPyV, MCPyV, HPyV6, HPyV7, HSV, EBV, CMV, HHV-6, HAdV and B19V). TTV was detected in 48% of the plasma samples available for testing, HPgV-1 in 28%, and the remaining viruses in 1% to 10%. After the infusion of stem cells, TTV was detected in half of the patients at D30 and was thereafter detected in almost all patients (prevalence ≥ 90% at M3, M6 and Y1). A peak prevalence was observed at D30 for BKPyV, CMV and HHV-6, reaching 41%, 27% and 19%, respectively. A peak of 27% was observed for HPyV6 at M3. In contrast, the curves of HPgV-1, JCPyV, MCPyV and HPyV7 were relatively flat with a prevalence ranging from 26% to 36%, 9% to 23%, 7% to 12%, and 0% to 12%, respectively (Table 4).

**Figure 3:**
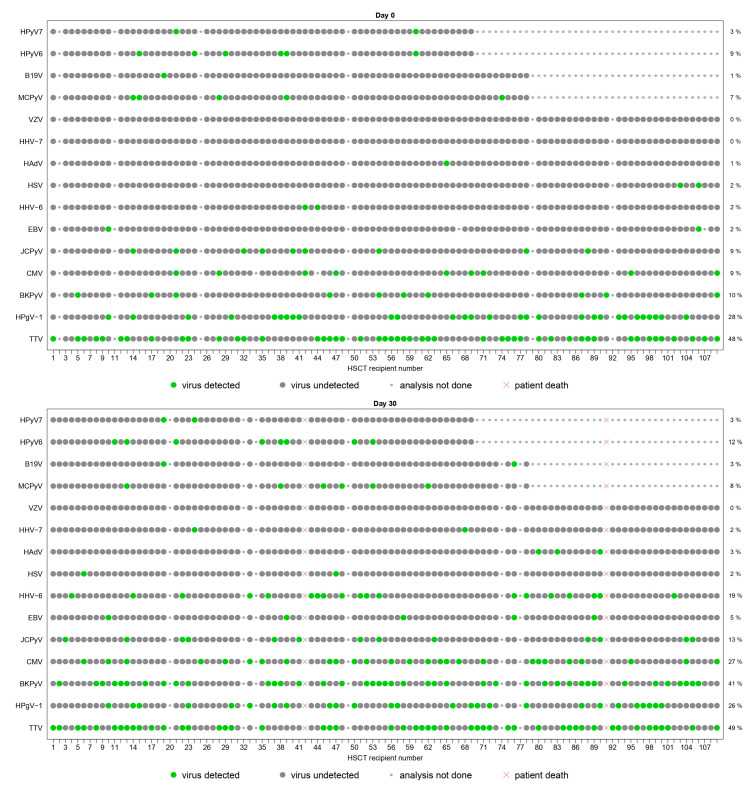

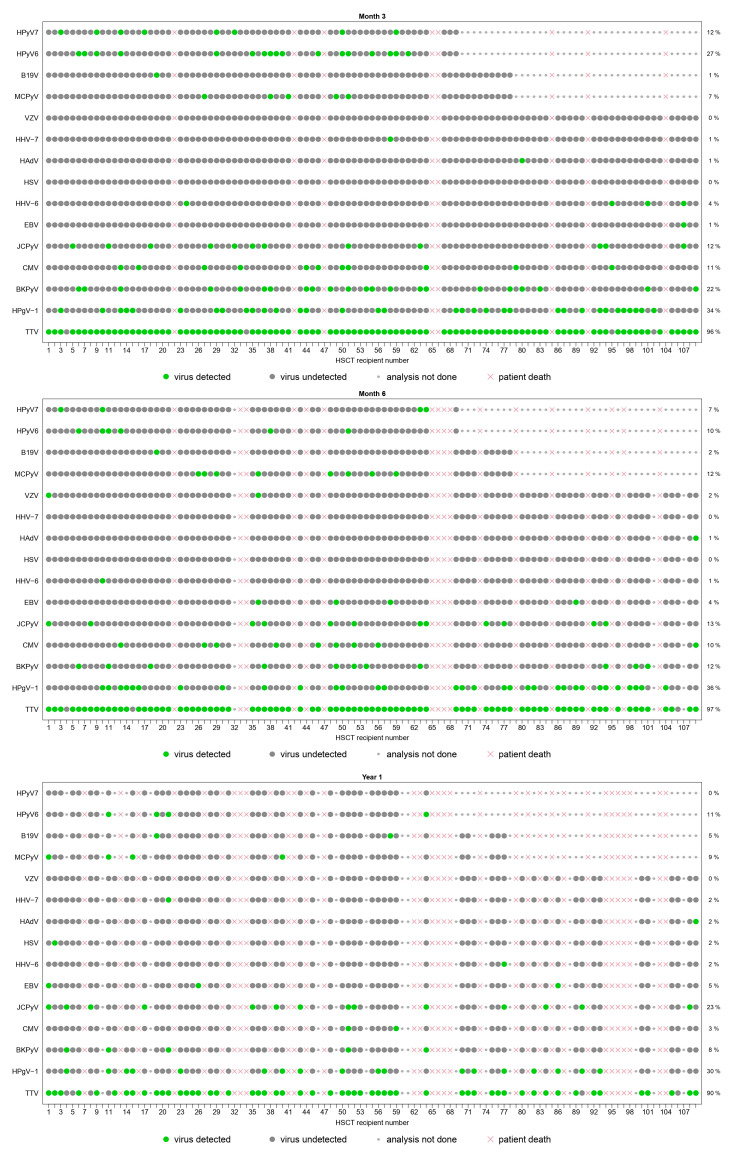
Schematic representation of the plasma detection of viral species screened from D0 to Y1.

Figure 3 represents individual virus detections at each time-point. Besides TTV and HPgV-1, seven viral species (CMV, HHV-6, BKPyV, JCPyV, MCPyV, HPyV6 and HPyV7) had a prevalence of ≥10% at one or more time-points. No patient had chromosomally integrated HHV-6. Four *Herpesviridae* (HSV, VZV, EBV and HHV-7) had prevalence ≤5% at all time-points. In particular, HSV, VZV and HHV-7 were detected in ≤2%, HAdV in ≤3% and B19V in ≤5% of the tested samples. Five (HPyV9, TSPyV, HBoV, EV and HPg-V2) viruses were never detected during follow-up.

The panels represent the plasma detection of viral species screened on plasma samples at day 0 (D0), day 30 (D30), month 3 (M3), month 6 (M6), year 1 (Y1). Each column represents one patient and the patients’ order stays the same throughout all graphs. Each line corresponds to one viral species. Green dots represent positive results of r(RT-)PCR assays. Grey dots represent negative results of r(RT-)PCR assays. The small grey dots represent analyses that were not performed due to the following reasons: viral species not included in the pre-specified group of testing, insufficient plasma sample volume, plasma sample not collected at the time of visit or missed visit. Red crosses represent analyses that were not performed due to patient’s death. All viral species are represented, except HPyV9, HPg-V2, HBoV, EV and TSPyV, which were never detected at any time-point.

#### 3.2.3. Plasma Viral Loads

**Figure 4:**
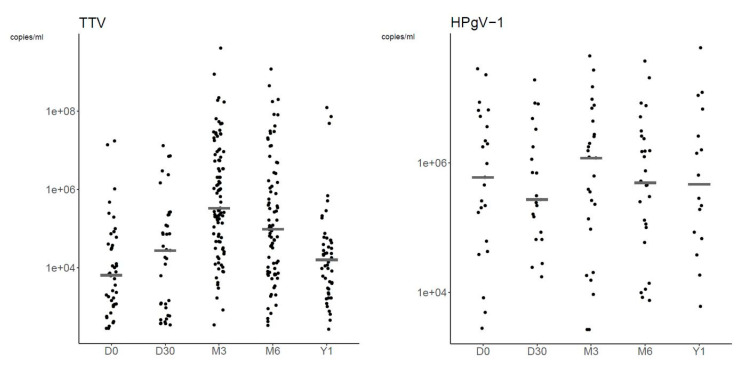
Dot plots of TTV and HPgV-1 plasma viral load at each time-point. The graphs are represented in a logarithmic scale to improve readability. Grey lines represent median plasma viral loads.

Figure 4 shows the plasma VLs of TTV and HPgV-1 at each time-point. TTV and HPgV-1 VLs peaked at M3 with a median VL of 3.29 × 10^5^ copies/mL (range, 3.37 × 10^2^ to 4.06 × 10^9^ copies/mL) and 1.18 × 10^6^ copies/mL (range, 2.61 × 10^3^ to 4.49 × 10^7^ copies/mL), respectively (Table 5).

We also analyzed the VLs of viruses at time-points which had a quantifiable VL in ≥5 patients. At D30, VLs were quantifiable for 16/27 (59.3%) and 13/19 (68.4%) patients with detectable CMV and HHV-6, respectively. Regarding *Polyomaviridae*: BKPyV VL was quantifiable in 5/41 (12.2%) positive patients at D30 with a median VL of 1.17 × 10^3^ copies/mL (range, 1.04 × 10^3^ to 1.41 × 10^3^ copies/mL); at M3, VLs of HPyV6 and HPyV7 were quantifiable in 7/17 (41.2%) and 5/8 (62.5%) positive patients, respectively, with median VLs of 1.3 × 10^3^ copies/mL (range, 2.64 × 10^2^ to 7.68 × 10^3^ copies/mL) and 1.63 × 10^3^ copies/mL (range, 2.73 × 10^2^ to 1.71 × 10^4^ copies/mL); and at M6, JCPyV VLs were quantifiable in 8/12 (66.7%) patients with a median VL of 1.17 × 10^3^ copies/mL (range, 1.34 × 10^2^ to 2.76 × 10^3^ copies/mL).

#### 3.2.4. Co-Detections

**Figure 5:**
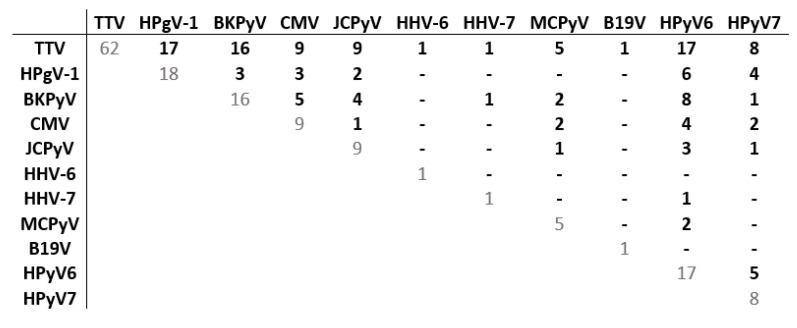
Matrix of dual co-detections among patients of group 3 at M3. The matrix represents dual co-detections among the 63 patients of group 3 (20 viral species screened) with at least one virus detected at M3. Each number represents the number of patients for whom the two viruses were detected (other viral species possibly co-detected are not shown). Abbreviations: CMV: cytomegalovirus; HHV-6: human herpes virus 6; HHV-7: human herpes virus 7; JCPyV: JC polyomavirus; BKPyV: BK polyomavirus; MCPyV: Merkel cell polyomavirus; HPyV6: human polyomavirus 6; HPyV7: human polyomavirus 7; B19V: parvovirus B19; TTV: torque teno virus; HPgV-1: human pegivirus 1.

Co-detections of ≥2 viruses were frequent (Figure 3). When all 20 viruses were screened, ≥2 viruses and ≥4 viruses were detected in ≥50% and ≥10% of patients after transplantation (from D30 to Y1), respectively (Table 6). The proportion of patients with co-infections was the highest at M3 (71.8%), with diverse types of co-detections observed, including TTV, HPgV-1 and several *Polyomaviridae.* Considering the multitude of combinations (Table S3), we specifically analyzed dual co-detections (Figure 5). TTV was most frequently co-detected with HPgV-1 and HPyV6 (27.0% of patients), BKPyV (25.4%), CMV and JCPyV (14.3%), and HPyV7 (12.7%). BKPyV and HPyV6 were co-detected in 12.7% of patients.

## 4. Discussion

In this longitudinal cohort study, we describe the prevalence of seventeen DNA and three RNA viral species, their co-detections and VL using r(RT-)PCR assays on plasma samples of 109 adult allo-HSCT recipients during a one-year period after transplantation. Our goal was to apply a systematic screening approach, not guided by symptoms or current guidelines, and to use r(RT-PCR) assays to provide comprehensive results for clinicians. We describe not only viruses that are known to frequently cause infections following HSCT, but also those that are usually not routinely screened for, as well as viruses that potentially cause unrecognized systemic infections.

Our study confirms that TTV and HPgV-1 infections are highly prevalent and could lead to prolonged infections at one year after allo-HSCT. Our results confirm that TTV infection occurs almost universally, often with sustained viremia at one year after allo-HSCT [4,11,12,13,14]. *Anelloviridae* are major and highly diverse components of the blood virome, causing chronic infections [15]. Their association with clinical manifestations remains unclear, although TTV may represent a biomarker for immune reconstitution and predictor of outcomes after allo-HSCT [11,15,16,17,18,19]. As previously reported, TTV was detected in almost 50% of patients before transplantation, with prevalence and VLs increasing at M3 [11,12,13,14,18]. Data are conflicting regarding these kinetics with some studies reporting a parallel increase of TTV VL and absolute lymphocyte count after HSCT, and others demonstrating a correlation between T-cell function and TTV viremia independently of T-cell count [11,12,16,18,19,20,21].

As reported by our group and others, HPgV-1 detection is frequent with persistent infections up to one year after HSCT [3,4,22,23]. Since HPgV-1 is known to cause chronic infections, HPgV-1 detection at multiple consecutive time-points in most patients suggests sustained viremia despite the long intervals between the sampling time-points. Besides a possible association with lymphoma, HPgV-1 has not been associated with any overt clinical manifestations after transplantation, despite complex interactions with the immune system [4,24,25,26,27,28,29]. Although lacking statistical significance, higher rates of progression-free survival and GVHD-free relapse-free survival, and lower aGVHD of grade ≥2 and relapse, have been reported among HPgV-1-infected versus HPgV-1-negative patients, illustrating that HPgV-1 may not be a silent bystander [3].

Our systematic screening strategy revealed that several *Polyomaviridae*, and in particular novel *Polyomaviridae* (MCPyV, HPyV6, HPyV7) that are overlooked in clinical routine, are frequently detected compared to classical culprits such as EBV, CMV, HHV-6 and HAdV.

The BKPyV viremia kinetics, with a D30 peak and lower levels at Y1 post-transplantation was in line with other studies that demonstrated viremia in up to 55% of patients, mostly during the first 100 days [4,6,30]. Despite progressive multifocal leukoencephalopathy being rare after HSCT, JCPyV viremia is common amongst HSCT recipients [4,5]. We observed an increasing prevalence with time, reaching 23% at Y1, contrary to another study reporting decreasing prevalence [31]. Several patients had multiple consecutive samples and among patients with JCPyV positive samples at M6, 67% had quantifiable and high VLs. These results suggest sustained JCPyV viremia and require further investigation. HPyV6 and HPyV7 were frequently detected at M3 with high VLs. Multiple HPyV6 positive samples with high VLs suggest HPyV6 sustained viremia. Their detection in HSCT-recipient blood has only been reported in one other study, where we described persistent HPyV6/7 viremia with similar VLs over months in 8% of allo-HSCT recipients with GVHD [5]. MCPyV detection was frequent, in line with another study using mNGS, where MCPyV DNA was detected in the plasma of 45% of allo-HSCT recipients at D30 [3]. In our cohort, most MCPyV positive samples had a VL below the LLOQ. Regarding MCPyV and other viruses detected at low levels, the systematic use of negative controls for all r(RT-)PCR experiments allowed for the exclusion of false positives.

From a clinical standpoint, the potential impact of these novel *Polyomaviridae* transient or sustained viremia as well as their interactions with the immune response remain unclear among HSCT recipients [4]. The question whether MCPyV, HPyV6 and HPyV7 are commensals viruses reactivating without negative effects during immunodeficiency remains open; we know that “asymptomatic” CMV reactivation, even with low VLs, is associated with deleterious effects beyond overt clinical events. Furthermore, the detection of multiple dsDNA viruses (EBV, CMV, HHV-6, HAdV, BKPyV) in blood after allo-HSCT is associated with overall mortality in a dose-dependent relationship [6]. Persistent high viremia of these viruses predicts mortality [7].

We observed CMV detection in up to 27% of patients at D30 with rates as low as 10% by M3 and M6, in contrast to our center’s previous report of 82.7% cumulative incidence of clinically significant CMV infection at 6 months [8]. This may be explained, in part, by the majority of patients developing CMV reactivation during the first weeks post-engraftment after D30 and receiving preemptive antiviral treatment leading to low rates of CMV reactivation at M3. CMV was frequently detected with other viral species, in particular TTV, BKPyV and HPgV-1 at M3. In a study that performed weekly screening of dsDNA viruses, the CMV detection rate was the highest at 6 weeks after HSCT, and CMV was mostly co-detected with BKPyV and HHV-6 [6]. HHV-6 reactivation is frequent after HSCT and may be associated with encephalitis among others [32]. HHV-6 early detection with a peak prevalence at D30 and VLs observed in our cohort are in line with previous studies [6,7,33,34,35]. Since HHV-6 reactivation frequently occurs early after transplantation, our sampling schedule may have missed some early reactivations [6,7,36]. The rapid decline in HHV-6 prevalence after D30 echoes that of CMV, likely due to the agents given for CMV prophylaxis with activity against HHV-6. The higher frequency of BKPyV, CMV and HHV-6 by D30, around the time of engraftment, and rapid decline thereafter may suggest associations with count recovery, immune reconstitution and other variables and requires further investigation.

HSV and VZV detection was rare, reflecting the efficacy of antiviral prophylaxis. EBV reactivation was rare and varies widely among centers according to transplant type, sample type and screening strategies [6,37,38]. HHV-7 was detected in up to 2% of patients, similar to previous results [3]. Its pathogenic role is less clear than HHV-6, but possible associations with central nervous system disease or hepatitis have been reported [4].

TSPyV, HPyV9, HBoV, HPgV-2 and EV were never detected. TSPyV is associated with a skin disease, mostly among SOT recipients, and viremia was reported in 1.9% of pediatric HSCT recipients [4,39]. HPyV9 has not been detected in HSCT recipient blood [3,4,5]. HBoV1 and HBoV2-4 are associated with respiratory and gastrointestinal diseases [4,40,41,42,43], and viremia occurs in up to 50% of symptomatic patients [4,44,45]. The clinical significance of HPgV-2, which may cause chronic infections, remains unknown [4,46,47]. EV viremia is extremely rare and occurs in 0.4% of pediatric HSCT recipients [48]. From a clinical standpoint, a syndromic approach may be favored for some viruses such as HBoV, TSPyV and EV.

Co-detections were particularly frequent at D30 and M3. Similarly, we reported the detection of ≥2 viral species in the plasma of 65% of patients 30 days after allo-HSCT [3], and the detection of ≥3 viral species in 64% of patients experiencing steroid-refractory/dependent GVHD [5]. GVHD and immunosuppressive treatments may have influenced the number of co-infections. In our study, almost 80% of patients suffered from GVHD one year after HSCT. These patients are at high risk for numerous viral infections due to the immune dysregulation of GVHD itself and immunosuppressive treatments [49,50,51,52,53,54,55].

Our study’s limitations include the size of the cohort and the large time intervals between testing. However, more frequent testing was not feasible considering the sampling design of the monocentric cohort of HSCT recipients and other constraints. Hence, as in the case of CMV, the prevalence of viral infections with transient and/or rare viremia may have been underestimated. Furthermore, due to the limited volume of each sample, we were unable to screen for astroviruses, which are associated with disseminated infections among immunocompromised patients [4,5,56,57,58]. Corticosteroid treatments as well as antiviral prophylaxis/treatments were not systematically recorded, although institutional strategies were unchanged. Antivirals such as (val-)aciclovir, (val-)gangiclovir or foscarnet may have influenced *Herpesviridae* viremia [59]; cidofovir may have influenced *Herpesviridae*, HAdV, BKPyV and potentially other *Polyomaviridae* detection [60,61,62,63]. The screening of donors and multiple blood products was not performed (except for routine pre-transplantation screening) but may be relevant to potential transmission events. In particular, some of the viruses screened here have been detected in blood products and stem cells [4]. Finally, in this descriptive study, we did not report clinical manifestations occurring at the time of viruses detection. Further studies are required to investigate the potential clinical manifestations and the impact on clinical outcomes of some of these viral infections after transplantation to fill the knowledge gap.

## 5. Conclusions

This study helps define the landscape and timing of several viral infections/reactivations, including many that are not systematically screened after allo-HSCT. Our results call for further studies investigating novel DNA and RNA virus pathogenicity after transplantation as well as the association between immune reconstitution or clinical outcomes and several novel *Polyomaviridae*/HPgV-1. From a diagnostic stewardship point of view, beyond the routinely screened viruses, a step-by-step approach further considering other assays targeting the multiple viral species screened in our study appears necessary. This study may contribute to revising diagnostic stewardship priorities and could represent a primer to assess the place of mNGS in selected cases.

## Figures and Tables

**Table 1:** Patients’ characteristics (109 allo-HSCT recipients).

	Total N = 109
**Demographics** Sex (male), *n* (%)	72 (66)
Age, median (IQR)	56 (18)
**Transplant source, *n* (%)**	
Peripheral blood cells	96 (88)
Bone marrow	13 (12)
**Underlying disease, *n* (%)**	
Acute myeloid leukemia	59 (54)
MDS/MDPS	27 (25)
Acute lymphoid leukemia	10 (9)
Myeloproliferative syndrome	4 (4)
Lymphoma	4 (4)
Chronic lymphocytic leukemia	2 (2)
Myeloma	2 (2)
Chronic myelogenous leukemia	1 (1)
Disease risk index *, *n* (%)	
Low	5 (5)
Intermediate	71 (65)
High	29 (27)
Very high	4 (4)
**EBMT risk score, *n* (%)**	
1	5 (5)
2	10 (9)
3	46 (42)
4	19 (17)
5	19 (17)
6	10 (9)
**EBV donor/recipient constellation, *n* (%)**	
+/+	96 (88)
−/+	8 (7)
+/−	3 (3)
−/−	2 (2)
**CMV donor/recipient constellation, *n* (%)**	
+/+	46 (42)
+/−	10 (9)
−/−	40 (37)
−/+	13 (12)
**Conditioning regimen, *n* (%)**	
Reduced intensity conditioning	74 (68)
Myeloablative conditioning	43 (39)
**Ex vivo T-cell depletion, *n* (%)**	19 (17)
**Donor type, *n* (%)**	
HLA identical sibling donor	28 (26)
HLA matched unrelated donor	47 (43)
Haploidentical donor	24 (22)
HLA mismatched unrelated donor	10 (9)

IQR: interquartile range; allo-HSCT: allogeneic hematopoietic stem cell transplantation; MDS/MDPS: myelodysplasic syndrome/myelodysplasic proliferative syndrome; +: seropositive; −: seronegative. * Disease risk index was defined according to Armand et al. [10].

**Table 2:** Number of patients in groups 1, 2, and 3 for which virological testing was performed at each time-point.

	Patients, N				
	Day 0	Day 30	Month 3	Month 6	Year 1
Group 1 (13 virus)	103	102	101	89	64
Group 2 (17 virus)	74	72	73	66	50
Group 3 (20 virus)	65	65	64	58	44
Missing data *	6	7	8	20	45

* Missing data are due to the following reasons: insufficient volume of plasma, plasma sample not collected at the time of visit, missed visit, or patient’s death.

**Table 3:** Period prevalence (D0 to Y1) of 20 viruses in plasma samples of 109 HSCT recipients.

Viral Specie	Viral Species Detected N (%)	Viral Species Undetectedor Not Tested N (%)
TTV	104 (95)	5 (5)
BKPyV	57 (52)	52 (48)
HPgV-1	51 (47)	58 (53)
JCPyV	41 (38)	68 (62)
CMV	36 (33)	73 (67)
HHV-6	26 (24)	83 (76)
HPyV6	26 (24)	83 (76)
MCPyV	23 (21)	86 (79)
HPyV7	15 (14)	94 (86)
EBV	12 (11)	97 (89)
HSV	5 (5)	104 (95)
HAdV	5 (5)	104 (95)
HHV-7	4 (4)	105 (96)
B19V	3 (3)	106 (97)
VZV	2 (2)	107 (98)
HPyV9	-	109 (100)
HPgV-2	-	109 (100)
HBoV	-	109 (100)
EV	-	109 (100)
TSPyV	-	109 (100)

**Table 4:** Point prevalence of 20 viruses in plasma samples of 109 HSCT recipients.

Viral Species	r(RT-)PCR Result	Time-Point				
		Day 0	Day 30	Month 3	Month 6	Year 1
**TTV**	**detected**	**49 (48%)**	**49 (49%)**	**97 (96%)**	**86 (97%)**	**55 (90%)**
	not detected	54 (52%)	52 (51%)	4 (4%)	3 (3%)	6 (10%)
	NA	6	8	8	20	48
**HPgV-1**	**detected**	**29 (28%)**	**26 (26%)**	**34 (34%)**	**32 (36%)**	**18 (30%)**
	not detected	74 (72%)	75 (74%)	67 (66%)	57 (64%)	43 (70%)
	NA	6	8	8	20	48
**BKPyV**	**detected**	**10 (10%)**	**41 (41%)**	**22 (22%)**	**11 (12%)**	**5 (8%)**
	not detected	93 (90%)	60 (59%)	79 (78%)	78 (88%)	56 (92%)
	NA	6	8	8	20	48
**JCPyV**	**detected**	**9 (9%)**	**13 (13%)**	**12 (12%)**	**12 (13%)**	**14 (23%)**
	not detected	94 (91%)	88 (87%)	89 (88%)	77 (87%)	47 (77%)
	NA	6	8	8	20	48
**MCPyV**	**detected**	**5 (7%)**	**6 (8%)**	**5 (7%)**	**8 (12%)**	**4 (9%)**
	not detected	69 (93%)	65 (92%)	68 (93%)	58 (88%)	39 (91%)
	NA	35	38	36	43	66
**HPyV6**	**detected**	**6 (9%)**	**8 (12%)**	**17 (27%)**	**6 (10%)**	**4 (11%)**
	not detected	59 (91%)	56 (88%)	47 (73%)	52 (90%)	34 (89%)
	NA	44	45	45	51	71
**HPyV7**	**detected**	**2 (3%)**	**2 (3%)**	**8 (12%)**	**4 (7%)**	**0 (0%)**
	not detected	63 (97%)	62 (97%)	56 (88%)	54 (93%)	38 (100%)
	NA	44	45	45	51	71
**CMV**	**detected**	**9 (9%)**	**27 (27%)**	**11 (11%)**	**9 (10%)**	**2 (3%)**
	not detected	92 (91%)	74 (73%)	90 (89%)	80 (90%)	59 (97%)
	NA	8	8	8	20	48
**HHV-6**	**detected**	**2 (2%)**	**19 (19%)**	**4 (4%)**	**1 (1%)**	**1 (2%)**
	not detected	101 (98%)	82 (81%)	97 (96%)	88 (99%)	60 (98%)
	NA	6	8	8	20	48
**HSV-1/2**	**detected**	**2 (2%)**	**2 (2%)**	**0 (0%)**	**0 (0%)**	**1 (2%)**
	not detected	101 (98%)	99 (98%)	101 (100%)	89 (100%)	60 (98%)
	NA	6	8	8	20	48
**VZV**	**detected**	**0 (0%)**	**0 (0%)**	**0 (0%)**	**2 (2%)**	**0 (0%)**
	not detected	103 (100%)	101 (100%)	101 (100%)	87 (98%)	61 (100%)
	NA	6	8	8	20	48
**EBV**	**detected**	**2 (2%)**	**5 (5%)**	**1 (1%)**	**4 (4%)**	**3 (5%)**
	not detected	99 (98%)	96 (95%)	100 (99%)	85 (96%)	58 (95%)
	NA	8	8	8	20	48
**HHV-7**	**detected**	**0 (0%)**	**2 (2%)**	**1 (1%)**	**0 (0%)**	**1 (2%)**
	not detected	103 (100%)	99 (98%)	100 (99%)	89 (100%)	60 (98%)
	NA	6	8	8	20	48
**HAdV**	**detected**	**1 (1%)**	**3 (3%)**	**1 (1%)**	**1 (1%)**	**1 (2%)**
	not detected	102 (99%)	98 (97%)	100 (99%)	88 (99%)	60 (98%)
	NA	6	8	8	20	48
**B19V**	**detected**	**1 (1%)**	**2 (3%)**	**1 (1%)**	**1 (2%)**	**2 (5%)**
	not detected	73 (99%)	69 (97%)	72 (99%)	65 (98%)	41 (95%)
	NA	35	38	36	43	66
**TSPyV**	**detected**	**0 (0%)**	**0 (0%)**	**0 (0%)**	**0 (0%)**	**0 (0%)**
	not detected	65 (100%)	64 (100%)	64 (100%)	58 (100%)	38 (100%)
	NA	44	45	45	51	71
**HPyV9**	**detected**	**0 (0%)**	**0 (0%)**	**0 (0%)**	**0 (0%)**	**0 (0%)**
	not detected	103 (100%)	101 (100%)	101 (100%)	89 (100%)	61 (100%)
	NA	6	8	8	20	48
**HBoV**	**detected**	**0 (0%)**	**0 (0%)**	**0 (0%)**	**0 (0%)**	**0 (0%)**
	not detected	74 (100%)	71 (100%)	73 (100%)	66 (100%)	43 (100%)
	NA	35	38	36	43	66
**HPgV-2**	**detected**	**0 (0%)**	**0 (0%)**	**0 (0%)**	**0 (0%)**	**0 (0%)**
	not detected	103 (100%)	101 (100%)	101 (100%)	89 (100%)	61 (100%)
	NA	6	8	8	20	48
**EV**	**detected**	**0 (0%)**	**0 (0%)**	**0 (0%)**	**0 (0%)**	**0 (0%)**
	not detected	74 (100%)	71 (100%)	73 (100%)	66 (100%)	43 (100%)
	NA	35	38	36	43	66

Viral species are classified by order of prevalence, as described in the manuscript. Unavailable data correspond to missing data and are due to the following reasons: insufficient volume of plasma, plasma sample not collected at the time of visit, missed visit or patient’s death. Abbreviations: HSV-1/2: herpes simplex virus 1/2; VZV: varicella zoster virus; EBV: Epstein–Barr virus; CMV: cytomegalovirus; HHV-6: human herpes virus 6; HHV-7 human herpes virus-7; JCPyV: JC polyomavirus; BKPyV: BK polyomavirus; MCPyV: Merkel cell polyomavirus; HPyV6: human polyomavirus 6; HPyV7: human polyomavirus 7; TSPyV: trichodysplasia spinulosa-associated polyomavirus; HPyV9: human polyomavirus 9; HAdV: human adenovirus; B19V: parvovirus B19; HBoV: human bocavirus; TTV: torque teno virus; EV: enterovirus; HPgV-1: human pegivirus 1; HPgV-2: human pegivirus 2; NA: not available.

**Table 5:** Plasma viral load of viral species detected in ≥5 patients at any time point.

			Plasma Viral Load *	
Time-Point	Viral Species	Patients N	Median (IQ)	Range
Day 0	TTV	42	6.35 × 10^3^ (1.15 × 10^3^–3.92 × 10^4^)	2.76 × 10^2^–1.73 × 10^7^
	HPgV-1	23	5.96 × 10^5^ (1.16 × 10^5^–4.39 × 10^6^)	2.79 × 10^3^–2.83 × 10^7^
Day 30	TTV	37	2.70 × 10^4^ (9.40 × 10^2^–2.19 × 10^5^)	3.39 × 10^2^–1.31 × 10^7^
	HPgV-1	21	2.71 × 10^5^ (8.39 × 10^4^–1.76 × 10^6^)	1.73 × 10^4^–1.89 × 10^7^
	CMV	16	2.02 × 10^2^ (1.18 × 10^2^–3.30 × 10^2^)	5.8 × 10^1^–5.70 × 10^3^
	HHV-6	13	1.16 × 10^3^ (8.42 × 10^2^–4.03 × 10^3^)	4.71 × 10^2^–9.88 × 10^3^
	BKPyV	5	1.17 × 10^3^ (1.11 × 10^3^–1.21 × 10^3^)	1.04 × 10^3^–1.41 × 10^3^
Month 3	TTV	93	3.29 × 10^5^ (4.66 × 10^4^–6.65 × 10^6^)	3.37 × 10^2^–4.06 × 10^9^
	HPgV-1	27	1.18 × 10^6^ (1.15 × 10^5^–3.60 × 10^6^)	2.61 × 10^3^–4.49 × 10^7^
	HPyV6	7	1.3 × 10^3^ (4.53 × 10^2^–2.59 × 10^3^)	2.64 × 10^2^–7.68 × 10^3^
	HPyV7	5	1.63 × 10^3^ (1.10 × 10^3^–5.39 × 10^3^)	2.73 × 10^2^–1.71 × 10^4^
Month 6	TTV	79	9.58 × 10^4^ (7.74 × 10^3^–1.57 × 10^6^)	3.33 × 10^2^–1.18 × 10^9^
	HPgV-1	28	4.90 × 10^5^ (1.10 × 10^5^–2.41 × 10^6^)	7.45 × 10^3^–3.74 × 10^7^
	JCPyV	8	1.17 × 10^3^ (4.21 × 10^2^–1.62 × 10^3^)	1.34 × 10^2^–2.76 × 10^3^
Year 1	TTV	48	1.58 × 10^4^ (2.92 × 10^3^–4.97 × 10^4^)	2.62 × 10^2^–1.23 × 10^8^
	HPgV-1	16	4.64 × 10^5^ (8.05 × 10^4^–3.61 × 10^6^)	6.01 × 10^3^–5.98 × 10^7^

IQ: interquartile; TTV: torque teno virus; HPgV-1: human pegivirus 1; CMV: cytomegalovirus; HHV-6: human herpesvirus 6; BKPyV: BK polyomavirus; HPyV6: human polyomavirus 6; HPyV7: human polyomavirus 7; JCPyV: JC polyomavirus. * Plasma viral loads are expressed in copies/mL, except for CMV for which plasma viral loads are expressed in IU/mL.

**Table 6:** Number of viral species detected in plasma samples at each time-point.

Number of Viral Species Detected	Patients, N (%)				
	Day 0 (N = 65)	Day 30 (N = 65)	Month 3 (N = 64)	Month 6 (N = 58)	Year 1 (N = 44)
0	15 (23.1%)	6 (9.2%)	1 (1.6%)	1 (17.2%)	1 (2.3%)
1	23 (35.4%)	20 (30.8%)	17 (26.6%)	20 (34.5%)	15 (34.1%)
2	18 (27.7%)	12 (18.5%)	23 (35.4%)	21 (36.2%)	13 (29.5%)
3	5 (7.7%)	18 (27.7%)	14 (21.9%)	8 (13.8%)	4 (9.1%)
4	1 (1.5%)	7 (10.8%)	5 (7.8%)	6 (10.3%)	5 (11.4%)
5	0	0	2 (3.1%)	2 (3.5%)	0
6	0	1 (1.5%)	2 (3.1%)	0	0
Missing data *	3 (4.6%)	1 (1.5%)	0	0	6 (13.6%)

Only patients with the 20 viral species were considered. * Missing data are due to the following reasons: insufficient volume of plasma, plasma sample not collected at the time of visit, missed visit or patient’s death.

## Data Availability

The datasets generated and/or analyzed during the current study are available in the Dryad repository [DOI:10.5061/dryad.5qfttdz81].

## Supplementary Materials

The following supporting information can be downloaded at: https://www.mdpi.com/article/10.3390/v15040928/s1, Table S1: DNA and RNA viral species screened in plasma samples of adult allo-HSCT recipients with specific qualitative and/or quantitative r(RT)-PCR assays. Table S2: Detailed list of the r(RT-)PCR used in the study. Table S3: Co-detections and their prevalence among the 63 patients of group 3. References [64,65,66,67,68,69,70,71,72,73,74,75,76,77] are cited in Supplementary Materials.
