# Supplementary material for: Longitudinal Detection of Twenty DNA and RNA Viruses in Allogeneic Hematopoietic Stem Cell Transplant Recipients Plasma
Source: Viruses. 2023 Apr 7;15(4):928. doi: 10.3390/v15040928 (PMC10142697; doi:10.3390/v15040928)
Supplement: Supplementary file 1 [file viruses-15-00928-s001.zip › viruses-2247529-supplementary.pdf]

Supplementary materials

Table S1: DNA and RNA viral species screened in plasma samples of adult allo-HSCT recipients with specific qualitative and/or quantitative r(RT)-PCR assays.

**DNA virus**	**RNA virus**
Herpesviridae	***Picornaviridae***
Herpes simplex virus (HSV) 1/2	Enterovirus (EV)
Varicella zoster virus (VZV)	***Flaviviridae***
Epstein.Barr virus (EBV)	Human pegivirus 1 (HPgV-1)
Cytomegalovirus (CMV)	Human pegivirus 2 (HPgV-2)
Human herpesvirus 6 (HHV-6)	
Human herpes virus 7 (HHV-7)	
Polyomaviridae	
JC polyomavirus (JCPyV)	
BK polyomavirus (BKPyV)	
Merkel cell polyomavirus (MCPyV)	
Human polyomavirus 6 (HPyV6)	
Human polyomavirus 7 (HPyV7)	
Trichodysplasia spinulosa-associated polyomavirus (TSPyV)	
Human polyomavirus 9 (HPyV9)	
Adenoviridae	
Human adenovirus (HAdV)	
Parvoviridae	
Parvovirus B19 (B19V)	
Human bocavirus (HBoV)	
Anelloviridae	
Torque teno virus (TTV)	

Table S2: Detailed list of the r(RT-)PCR used in the study.

Virus detected	Assay	Elution volume used [uL]	Sequence (5' to 3')			Final [uM] Fwd/Rev/Probe	Lower limit of quantification	Reference
			Fwd primer	Probe	Rev Primer			
**Epstein Barr virus**	EBV	Not available	Not available	Not available	Not available	Not available	5.0E2 copies/ml	artus EBV QS-RGQ kit (Qiagen) /cobas® EBV (Roche)
**Cytomegalovirus**	CMV	Not available	Not available	Not available	Not available	Not available	5.6E1 IU/ml	RealTime CMV assay (Abbott) /cobas® CMV (Roche)
**Herpes simplex virus 1/2**	HSV-1/2 screening	5	HSV-F:<br>CCGTCAGCACCTTCATCGA	HSVgb:<br>FAM CCACGAGATCAAGGACAGCGGCC TAMRA	HSV-R:<br>CGCTGGACCTCCGTGTAGTC	300/300/100	Not quantitative	Aubert M et al. [1]
	HSV-1/2 typing	5	GbTypF:<br>CGCATCAAGACCACCTCCTC	GbTyp1:<br>VIC TGGCAACGCGGCCCAAC TAMRA	GbTypR:<br>GCTCGCACCACGCGA	900/600/100 (each probe)	2.6E3 copies/ml	Corey L et al. [2]
		5		GbTyp2:<br>FAM CGGCGATGCGCCCCAG TAMRA			2.6E2 copies/ml	
**Varicella Zoster virus**	VZV	5	VZV UP:<br>CGGCATGGCCCGTCTAT	VZV P:<br>FAM ATTCAGCAATGGAAACACACGACGCC TAMRA	VZV DP:<br>TCGCGTGCTGCGGC	600/600/200	2.6E2 copies/ml	Weidmann M et al. [3]
**Human herpesvirus 6**	HHV-6 screening	5	HHV-6FORTAQ1:<br>GACAATCACATGCCTGGATAATG	HHV-6PROBE:<br>FAM AGCAGCTGGCGAAAAGTGCTGTGC TAMRA	Taq2 RevHHV-6a+b:<br>TGTAAGCGTGTGGTAATGGACTAA	600/600/200	Not applicable	Gautheret-Dejean A et al. [3]
	HHV-6 quantitative	10	Not available	Not available	Not available	Not available	3.5E2 copies/ml	Human Herpes virus 6 kit (Genesig)
**Human herpesvirus 7**	HHV-7	10	Not available	Not available	Not available	Not available	2.6E3 copies/ml	Human Herpes Virus 7 genomes kit (Genesig)
**JC polyomavirus**	JCPyV screening	5	JCT-3:<br>AGTGTTGGGATCCTGTGTTTTCA<br><br>JRR-1:<br>GGAGCCCTGGCTGCAT	JCT-1.2:<br>FAM CATCACTGGCAAACAT NFQ MGB<br><br>JRR-1.1:<br>VIC CTGGCAGTTATAGTGAAAC NFQ MGB	JCT-4:<br>GTGGGATGAAGACCTGTTTTGC<br><br>JRR-2:<br>TGTGATTAAGGACTATGGGAGG	each primer: 300<br>each probe: 200	Not quantitative	Ryschkewitsch CF et a1. [3]
	JCPyV quantitative	10	Not available	Not available	Not available	Not available	1.25E2 copies/ml	JCV ELITe MGB kit (ELITechGroup)
**Human polyomavirus 9**	HPyV9	5	HPyV9-VP1-F:<br>CTAGGGAACAATTTGAATATCAGGAA	HPyV9-VP1-Prb:<br>FAM AAGTTAGGCTGAGGCGGGAGATAGGG BHQ1	HPyV9-VP1-R:<br>ATAGTGTCCAGATCTAGGCTCTGAAC	600/600/200	2.6E2 copies/ml	Rockett RJ et al. [4]
**Human adenovirus**	HAdV screening	5	AQ2 For:<br>GCCCCAGTGGTCTTACATGCACATC	AP:<br>FAM TGCACCAGACCCGGGCTCAGGTACTCCGA TAMRA	ADV Rev:<br>GVGCCACGGTGGGGTTTCTAAACTT	900/900/200	Not quantitative	Adapted from Verheyen J et al. [5]
	HAdV quantitative	10	Not available	Not available	Not available	Not available	2.0E2 IU/ml	Adenovirus R-gene kit (Argene)
**Torque teno virus**	TTV	5	TTV(Y)-F:<br>GTTTTCYACGCCCGTCC	TTV_probe:<br>FAM ACTCACCTHCGGCACCCGC BHQ1	TTV(Y)-R:<br>CCTTGACTYCGGTGTGTAA	900/900/200	2.5E2 copies/ml	Masouridi-Levrat S et al. [6]
**Human pegivirus 1 and 2**	HPgV-1	5	Pegivirussenseprimer:<br>GGCGACCGGCCAAAA	Pegivirusprobe:<br>FAM TGACCGGGATTTACGACCTACCAACCCT TAMRA	Pegivirusantisenseprimer:<br>CTTAAGACCCACCTATAGTGGCTACC	900/900/200	2.6E3 copies/ml	Chivero ET et al. [7]
	HPgV-2	5	HPgV-25UTR-F:<br>CGCTGATCGTGCAAAGGGATG	HPgV-25UTR-probe:<br>FAM GCACCACTCCGTACAGCCTGAT TAMRA	HPgV-25UTR-R:<br>GCTCCACGGACGTCACACTGG	600/600/200	2.6E2 copies/ml	Frankel M et al. [8]
**BK virus**	BK	10	Not available	Not available	Not available	Not available	5.0E2 copies/ml	BK Virus R-gene kit (Argene)
**CDV (internal control)**	CDV	5	CDV-for:<br>GCTACCCAAGAAACCGTCATTG	CDV-probe:<br>Yakima yellow CGTTCAGGGAGTCCAGGACTACGTCAAC BHQ1	CDV-rev:<br>GCATGGCAGGGACGAGTT	900/900/200	Not quantitative	Cordey S et al. [9]

**Bocavirus 1/2/3/4**	Bocavirus 1-4	5	HBoV1F:<br>CCTATATAAGCTGCTGCACTTCCTG<br><br>HBoV2-3-4F:<br>GCACTTCCGCATYTCGTCAG	HBoV1-4-probe:<br>FAM CCAGAGATGTTCACTCGCCG MGB-Eclipse®	HBoV1R:<br>AAGCCATAGTAGACTCACCACAAG<br><br>HBoV3R:<br>GTGGATTGAAAGCCATAATTTGA<br><br>HBoV2-4R:<br>AGCAGAAAAGGCCATAGTGTCA	each primer: 600<br>probe: 300	2.6E2 copies/ml	Kantola K et al. [10]
**Merkel Cell Polyomavirus**	MCPyV	5	MCPyV-Fwd:<br>CCCTTTGGAGCAAATTCCA	MCPyV-probe:<br>FAM CAAAATATCCACAAGCTCAGAAGTGA TAMRA	MCPyV-Rev:<br>CTGACCTCATCAAACATAGAGAA	600/600/250	2.6E2 copies/ml	Arvia R et al. [11]
**Enterovirus**	EV	5	Entero/Ge/08 Fwd:<br>GCTGCGYTGGCGGCC	Entero/Ge/08 pro:<br>FAM CTCCGGCCCCTGAATGYGGCTAA TAMRA	Entero/Ge/08 Rev:<br>GAAACACGGACACCCAAAGTAGT	900/900/200	6.E2 copies/ml	Tapparel C et al. [12]
**Parvovirus B19**	B19	10	Not available	Not available	Not available	Not available	5.0E2 IU/ml	Parvovirus B19 R-gene kit (Argene)
**Human polyomavirus 6**	HPyV6	5	HPyV6-VP2-F:<br>TTGAGGAGCTGGACAAAGAGATT	HPyV6-VP2-Prb:<br>FAM AGGAAGATGCCTTGTCACAGAAAAGGAAATG BHQ1	HPyV6-VP2-R:<br>TCTGGGAACTTTTGAATTGGT	600/600/200	2.6E2 copies/ml	Antonsson A et al. [13]
**Human polyomavirus 7**	HPyV7	5	HPyV7-VP2-F:<br>GAGGAAGGAAACACTCCCCAGTA	HPyV7-VP2-Prb:<br>FAM ACTATACCTCAATGGATGCTTTTTGT BHQ1	HPyV7-VP2-R:<br>TTCACTTCTTTTTGTAGCTCCTCAAG	900/900/250	2.6E2 copies/ml	Antonsson A et al. [13]
**Trichodysplasia spinulosa-associated polyomavirus**	TSPyV	5	TSPyVa-F:<br>TGGTGGATTCATGGCAGAAGAG	TSPyVa-probe:<br>FAM TCTGGGTCCTGCATGGTGTCAAA BHQ-1	TSPyVa-R:<br>GCTGGTGGGAAAGCTTCAATC	600/600/250	2.6E2 copies/ml	Urbano PR et al. [14]

Table S3: Co-detections and their prevalence among the 63 patients of group 3.

**Viral species detected**	**Patients, N (%)**
TTV	16 (25%)
HPgV-1	1 (2%)
TTV, HPgV-1	9 (14%)
TTV, BKPyV	3 (5%)
TTV, BKPyV, HPyV6	3 (5%)
TTV, JCPyV	3 (5%)
TTV, BKPyV, CMV	2 (3%)
TTV, BKPyV, JCPyV	2 (3%)
TTV, HPyV6	2 (3%)
TTV, HPyV6, HPyV7	2 (3%)
TTV, MCPyV	2 (3%)
TTV, BKPyV, HPyV6, MCPyV	1 (2%)
TTV, BKPyV, CMV, HPgV-1	1 (2%)
TTV, BKPyV, CMV, HPgV-1, HPyV6, HPyV7	1 (2%)
TTV, BKPyV, CMV, MCPyV, JCPyV, HPyV6	1 (2%)
TTV, CMV	1 (2%)
TTV, CMV, HPyV6	1 (2%)
TTV, CMV, MCPyV	1 (2%)
TTV, CMV, HPgV-1, HPyV6, HPyV7	1 (2%)
TTV, HHV-6	1 (2%)
TTV, BKPyV, HPyV6, HHV-7	1 (2%)
TTV, JCPyV, HPyV6, BKPyV, HPgV-1	1 (2%)
TTV, JCPyV, HPyV7	1 (2%)
TTV, JCPyV, HPyV6, HPgV-1	1 (2%)
TTV, HPyV7	1 (2%)
TTV, B19V	1 (2%)
TTV, HPgV-1, HPyV6	1 (2%)
TTV, HPgV-1, HPyV6, HPyV7	1 (2%)
TTV, HPgV-1, HPyV7	1 (2%)

The table describes the co-detections and their prevalence among the 63 patients of group 3 (20 viral species screened) with at least one virus detected at 3 months after allo-HSCT. No virus was detected in 1/64 patients of group 3 at month 3.

Abbreviations: CMV: cytomegalovirus; HHV-6: human herpes virus 6; HHV-7: human herpes virus 7; JCPyV: JC polyomavirus; BKPyV: BK polyomavirus; MCPyV: Merkel cell polyomavirus; HPyV6: human polyomavirus 6; HPyV7: human polyomavirus 7; B19V: parvovirus B19; TTV: torque teno virus; HPgV-1: human pegivirus 1.

References

1. Aubert, M.; Boyle, N.M.; Stone, D.; Stensland, L.; Huang, M.-L.; Magaret, A.S.; Galetto, R.; Rawlings, D.J.; Scharenberg, A.M.; Jerome, K.R. In vitro Inactivation of Latent HSV by Targeted Mutagenesis Using an HSV-specific Homing Endonuclease. *Mol. Ther. Nucleic Acids* **2014**, *3*, e146.
2. Corey, L.; Huang, M.L.; Selke, S.; Wald, A. Differentiation of herpes simplex virus types 1 and 2 in clinical samples by a real-time taqman PCR assay. *J. Med. Virol.* **2005**, *76*, 350–355.
3. Weidmann, M.; Meyer-Konig, U.; Hufert, F.T. Rapid detection of herpes simplex virus and varicella-zoster virus infections by real-time PCR. *J. Clin. Microbiol.* **2003**, *41*, 1565–1568.
4. Rockett, R.J.; Sloots, T.P.; Bowes, S.; O'Neill, N.; Ye, S.; Robson, J.; Whiley, D.M.; Lambert, S.; Wang, D.; Nissen, M.; et al. Detection of novel polyomaviruses, TSPyV, HPyV6, HPyV7, HPyV9 and MWPyV in feces, urine, blood, respiratory swabs and cerebrospinal fluid. *PLoS ONE* **2013**, *8*, e62764.
5. Verheyen, J.; Timmen-Wego, M.; Laudien, R.; Boussaad, I.; Sen, S.; Koc, A.; Uesbeck, A.; Mazou, F.; Pfister, H. Detection of adenoviruses and rotaviruses in drinking water sources used in rural areas of Benin, West Africa. *Appl. Environ. Microbiol.* **2009**, *75*, 2798–2801.
6. Masouridi-Levrat, S.; Pradier, A.; Simonetta, F.; Kaiser, L.; Chalandon, Y.; Roosnek, E. Torque teno virus in patients undergoing allogeneic hematopoietic stem cell transplantation for hematological malignancies. *Bone Marrow Transplant.* **2016**, *51*, 440–442.
7. Chivero, E.T.; Bhattarai, N.; Rydze, R.T.; Winters, M.A.; Holodniy, M.; Stapleton, J.T. Human pegivirus RNA is found in multiple blood mononuclear cells in vivo and serum-derived viral RNA-containing particles are infectious in vitro. *J. Gen. Virol.* **2014**, *95*, 1307–1319.
8. Frankel, M.; Forberg, K.; Coller, K.E.; Berg, M.G.; Hackett, J.; Cloherty, G.; Dawson, G.J. Development of a high-throughput multiplexed real time RT-PCR assay for detection of human pegivirus 1 and 2. *J. Virol. Methods.* **2017**, *241*, 34–40.
9. Cordey, S.; Junier, T.; Gerlach, D.; Gobbini, F.; Farinelli, L.; Zdobnov, E.M.; Winther, B.; Tapparel, C.; Kaiser, L. Rhinovirus genome evolution during experimental human infection. *PLoS ONE* **2010**, *5*, e10588.
10. Kantola, K.; Sadeghi, M.; Antikainen, J.; Kirveskari, J.; Delwart, E.; Hedman, K.; Söderlund-Venermo, M. Real-time quantitative PCR detection of four human bocaviruses. *J. Clin. Microbiol.* **2010**, *48*, 4044–4050.
11. Arvia, R.; Sollai, M.; Pierucci, F.; Urso, C.; Massi, D.; Zakrzewska, K. Droplet digital PCR (ddPCR) vs quantitative real-time PCR (qPCR) approach for detection and quantification of Merkel cell polyomavirus (MCPyV) DNA in formalin fixed paraffin embedded (FFPE) cutaneous biopsies. *J. Virol. Methods.* **2017**, *246*, 15–20.
12. Tapparel, C.; Cordey, S.; Van Belle, S.; Turin, L.; Lee, W.-M.; Regamey, N.; Meylan, P.; Mühlemann, K.; Gobbini, F.; Kaiser, L. New molecular detection tools adapted to emerging rhinoviruses and enteroviruses. *J. Clin. Microbiol.* **2009**, *47*, 1742–1749.
13. Antonsson, A.; Bialasiewicz, S.; Rockett, R.J.; Jacob, K.; Bennett, I.C.; Sloots, T.P. Exploring the prevalence of ten polyomaviruses and two herpes viruses in breast cancer. *PLoS ONE* **2012**, *7*, e39842.
14. Urbano, P.R.; Nali, L.H.; Bicalho, C.S.; Pierrotti, L.C.; David-Neto, E.; Pannuti, C.S.; Romano, C.M. New findings about trichodysplasia spinulosa-associated polyomavirus (TSPyV)--novel qPCR detects TSPyV-DNA in blood samples. *Diagn. Microbiol. Infect. Dis.* **2016**, *84*, 123–124.
